# Distant Multilevel Spinal Metastasis Secondary to Hypopharyngeal Squamous Cell Carcinoma

**DOI:** 10.7759/cureus.64715

**Published:** 2024-07-17

**Authors:** R. Parker Kirby, Sarah Kim, Lama M Abdurrahman, Alexander Kietzman, James Doan, David Hernandez

**Affiliations:** 1 Otolaryngology - Head and Neck Surgery, Baylor College of Medicine, Houston, USA; 2 Pathology, Baylor College of Medicine, Houston, USA

**Keywords:** head and neck cancer pathology, otolaryngology-head & neck surgeons, head and neck surgery with free flap reconstruction, spinal metastasis, head and neck squamous cell carcinoma (hnscc)

## Abstract

Head and neck squamous cell carcinomas account for most head and neck malignancies. While multi-modality treatment may be offered for locally advanced cancer, distant metastasis still occurs in a significant number of patients. This paper aims to present a rare case of a patient who developed bony metastases in the cervical spine from a primary hypopharyngeal malignancy status post-laryngopharyngectomy. We report a case of a male patient presenting with acute-on-chronic hypercapnic and hypoxic respiratory failure with two months of dysphagia and weight loss. On arrival, a barium swallow revealed mucosal irregularity of the upper thoracic esophagus as well as narrowing and stenosis. A direct laryngoscopy with biopsy revealed squamous cell carcinoma of the hypopharynx. CT neck and chest were obtained for staging. He underwent a total laryngopharyngectomy, bilateral neck dissections, and a free flap. His final staging was pT4aN2c cM0. Three months post-admission, during inpatient radiation therapy, the patient reported midline neck pain with focal bone tenderness, and an MRI was obtained of his cervical and thoracic spine with a report concerning spinal metastasis.A subsequent bone biopsy showed findings consistent with osseous metastasis from a primary hypopharyngeal squamous cell carcinoma. After multidisciplinary goals of care discussions, the patient ultimately decided to be discharged to inpatient hospice.

This report highlights a rare case of hypopharyngeal carcinoma metastasis to the cervical spine. Despite its rarity and poor prognosis, such a metastasis should be considered in the differential diagnosis of patients with a history of hypopharyngeal squamous cell carcinoma and localizing symptoms.

## Introduction

Head and neck squamous cell carcinomas (HNSCCs) arise from the mucosal epithelium of the upper aerodigestive tract and account for most head and neck malignancies, excluding well-differentiated thyroid cancer. The prevalence of HNSCCs varies globally, but their risk factors are well-established, as 75-85% of HNSCCs are attributed to tobacco use and alcohol consumption. Oropharyngeal cancer is increasingly associated with human papillomavirus (HPV) infection, especially HPV-16. FDA-approved vaccines covering HPV-16 and HPV-18 offer potential prevention for HPV-positive HNSCC [1,2].

HPV-negative oropharyngeal cancer behaves similarly to HNSCCs from other aerodigestive sites such as the oral cavity and the larynx and is primarily associated with tobacco use. No effective screening strategy exists, with early detection relying on physical examination. While some premalignant lesions progress to invasive cancer, many patients present with advanced-stage HNSCCs. Treatment involves surgical resection followed by adjuvant (chemo)radiation or radiation-based treatment (with chemotherapy typically given concurrently for advanced cases) [1,2].

We report a case of hypopharyngeal squamous cell carcinoma status post-laryngopharyngectomy that, while undergoing radiation therapy, developed spinal metastases. We share histopathological findings from pharyngeal and spinal biopsies obtained during our patient’s hospitalization.

## Case presentation

The patient is a 66-year-old male with a past medical history significant for alcoholic cirrhosis (42 drinks/week), tobacco use (35 pack years), COPD (forced expiratory volume in 1 second/functional vital capacity=67%, long-acting beta agonist/short-acting beta agonist daily, no history of exacerbations, home O_2_) and pulmonary hypertension, presenting with acute-on-chronic hypercapnic and hypoxemic respiratory failure and two months of dysphagia and weight loss (30 pounds). The patient was in his usual state of health until two months prior to presentation when he noticed progressive shortness of breath with a worsening chronic productive cough. Compared to baseline, he felt more fatigued, had decreased appetite, and noticed he was losing weight. His home O_2_ needs had increased. Hours before presenting to the emergency room, his O_2_ saturation was reported to be 54% (baseline 70-80%), and he had been getting gradually more confused, altered, and fatigued. 

**Figure 1:**
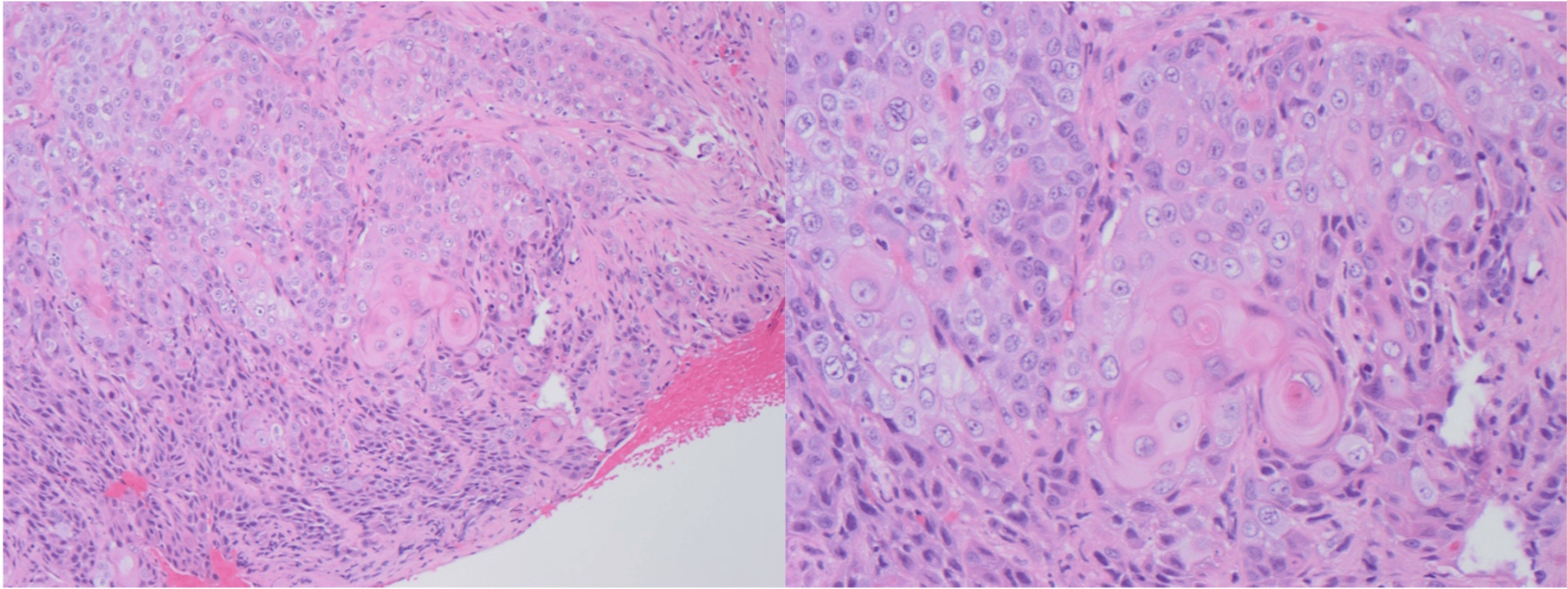
Hematoxylin and eosin (H&E) stained sections (100x) show the native larynx with infiltrating squamous cells forming sheets and nests. The cells have apparent cytoplasm and form “keratin pearls”.

Upon arrival at our institution, the patient was intubated and admitted to the medical ICU for acute-on-chronic hypoxemic respiratory failure. While the etiology was unclear at the time, the patient rapidly improved, was extubated within 24 hours, and was back to baseline home oxygen requirements. During the hospitalization, a modified barium swallow study was performed to evaluate his dysphagia, and it showed stenosis and mucosal irregularity of the upper thoracic esophagus. A subsequent esophagogastroduodenoscopy revealed a hypopharyngeal mass. Direct laryngoscopy with biopsy was performed, which confirmed a fungating mass overlying the supraglottic, glottic, right piriform, and postcricoid subsites, without esophageal extension. CT soft tissue of the neck showed a large predominantly right-sided hypopharyngeal and supraglottic mass, measuring greater than 4 cm with right thyroid cartilage invasion and tumor beyond the larynx staged at ​​cT4N2cM0. The patient underwent a total laryngopharyngectomy, bilateral neck dissections, and a free flap reconstruction. Pathology from the surgery showed a 5.5 cm tumor centered in the arytenoepiglottic fold, extending to the right pyriform sinus, bilateral posterior pharynx, bilateral hypopharynx, and right upper esophageal mucosa and muscularis propria. The mass abutted the thyroid cartilage, and both lymphovascular and perineural invasion were identified; only one lymph node was negative for metastatic carcinoma. Histology determined a squamous cell carcinoma (Figure 1). 

He was then started on IMRT radiation at 250 cGy in 20 fractions. He received 20 total doses of radiation over one month. His hospital course was complicated by MSSA bacteremia, requiring a pause in radiation therapy, and significant hypoxia required 6 L/min oxygen via trach collar throughout the stay.

**Figure 2:**
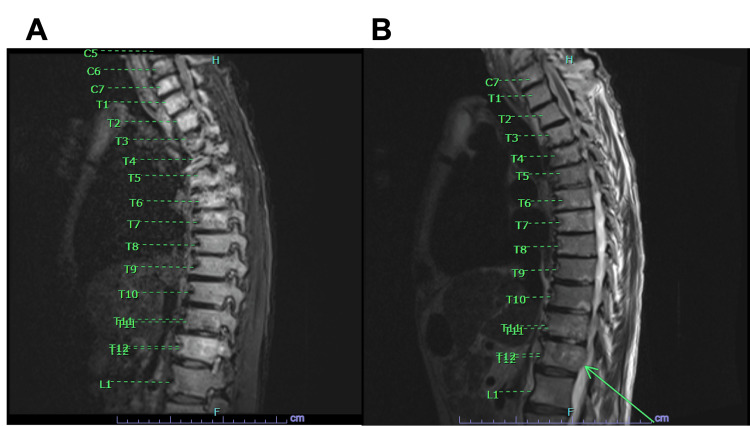
(A) A STIR MRI sequence of the lower cervical and thoracic spine. The radiologist noted diffuse marrow heterogeneity specifically in C7, T5, and T12 vertebral bodies. (B) A T2 weighted MRI demonstrating a sliver of epidural soft tissue along the right aspect of T12, noted with an arrow. No significant cord compression was observed, however. STIR: Short tau inversion recovery

Subsequently, our patient reported midline back pain, and there was concern for potential osteomyelitis from the MSSA pneumonia. An MRI of the thoracic and cervical spine showed diffuse marrow heterogeneity, with concern for extensive osseous metastatic disease involving the cervical and thoracic spine. It was noted to potentially involve C6, C7, T5, T6 and T12 vertebral bodies (Figures 2A, 2B). Other areas of focal marrow enhancement were seen throughout the spinal cord.

**Figure 3:**
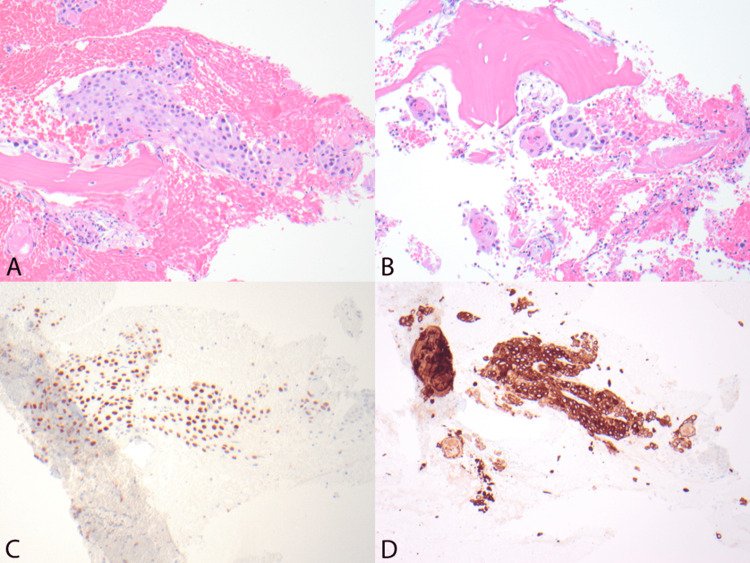
(A,B) Hematoxylin and eosin (H&E) stained sections (100x) show bone with infiltrating squamous cells forming sheets and nests. The cells have abundant cytoplasm, moderately pleomorphic nuclei, and form "keratin pearls" (B). (C, D) Immunohistochemical staining (100x) for P40 (C) and Cytokeratin 5/6 (D) show positive staining.

A biopsy was performed at the T12 vertebrae, showing evidence supporting a squamous cell carcinoma compatible with the patient’s known hypopharyngeal primary (Figures 3A-3D). Immunohistochemistry of the sample was positive for CK5/6 and p40. The patient then went on to receive a palliative course of XRT to the spine.

## Discussion

HNSCCs account for roughly 4.5% of cancer diagnoses and deaths worldwide, with 890,000 new cases and 450,000 deaths annually [3]. In 2024, there are 58,450 estimated new cases of HNSCCs and 12,230 estimated deaths, representing 2.0% of all new cancer deaths in the US [4].

Metastasis of HNSCCs is considered to occur in approximately 10%-20% of cases [5,6]. Metastases are most commonly identified in the regional cervical lymph nodes, but distant metastases can be seen in the lung, liver, and mediastinum with rare reports of sites identified in skeletal muscle [7], heart [8], and spinal cord [9]. Metastases to the bone have historically been thought to be a rare occurrence (estimated at ~1% of distant metastases), but recent literature suggests this is more common than previously understood (5%-10%) [10]. In cases of HNSCCs, bony metastasis to the lumbar spine, pelvis, shoulder, thorax, face, and extremities have been identified at rare, but reasonable, rates of approximately 4% [11]. However, the presence of hypopharyngeal squamous cell carcinoma metastasis to the cervical and thoracic spine is exceedingly rare and has yet to be documented with histologic confirmation.

In our patient, multiple bony metastases to the cervical and thoracic spine were identified radiographically and confirmed histopathologically. H&E-stained sections of the vertebral biopsy show bone with infiltrating squamous cells forming sheets and nests (Figure 2A). These cells have abundant cytoplasm, moderately pleomorphic nuclei, and form “keratin pearls” (Figure 2B), pathognomonic of SCCs. The subsequent IHC staining definitively identified these lesions as metastatic SCC by demonstrating stains positive for P40 (Figure 2C) and Cytokeratin (CK) 5/6. P40 is a nuclear marker with expression in squamous cell differentiation that is utilized for its superior specificity for SCC [12]. Furthermore, the lesion stains positive for CK 5 and 6 (Figure 2D), a marker highly predictive of primary tumor of squamous epithelial origin when differentiating metastatic carcinomas of unknown primary site [13].

Despite the rarity of bony metastasis from HNSCCs and regardless of the patient’s prognosis, metastasis should be considered in the differential diagnosis of patients with a history of hypopharyngeal SCC and localizing symptoms. Symptoms, as in our patient’s case, may include focal bone pain and tenderness. Localized pain and swelling could potentially limit the range of motion or even cause deformities. More obvious signs of bony metastases include pathologic fractures, spinal cord compression (motor or sensory deficits, bladder, or bowel incontinence), and radicular symptoms because of compression of spinal nerve roots in spinal metastases. The presence of clinical symptoms is often a significant factor in accurately narrowing the differential diagnosis [14]. However, the simple fact of the rarity of HNSCC metastasis to the bone may trigger attribution and availability heuristics, which in this case would have failed the clinician [15]. A history of chronic neck pain (secondary to past trauma, degenerative processes, etc.) would have further confounded the diagnostic schema and challenged clinical decision-making.

As a result of his newly confirmed diagnosis and its poor prognosis, and in congruence with the discussed goals of care, our patient received palliative XRT in accordance with the society recommendations of the American Society for Radiation Choosing Wisely guidelines [16]. Beyond palliative XRT, other therapeutic options for metastatic SCC exist for patients with better performance status and differing goals of care. The current standard of care for these patients is pembrolizumab/cisplatin/5-fluorouracil or pembrolizumab alone for PD-L1 positive tumors. Immunotherapy with immune checkpoint inhibitors or a combination of chemoimmunotherapy is an option as well [17]. Patients with metastatic HNSCCs that express programmed cell death protein 1 and its ligand (PD-L1) should be treated with pembrolizumab or the combination of pembrolizumab and the first-line chemotherapy mentioned above [18].

## Conclusions

This report is a rare account of cervical and thoracic bony spinal metastases from hypopharyngeal squamous cell carcinoma. New localizing symptoms of patients with a history of advanced HNSCCs should be given attention as they may represent pathologic recurrence of disease.
